# Decision making about infertility treatment: does unlimited access lead to inappropriate treatment?

**DOI:** 10.1186/s13584-016-0083-6

**Published:** 2016-06-14

**Authors:** Evan R. Myers

**Affiliations:** Department of Obstetrics & Gynecology and Duke Evidence-Synthesis Group, Duke University School of Medicine, 2400 Pratt Street, Durham, NC 27710 USA

## Abstract

The proportion of women aged 40–44 undergoing IVF treatment covered by Maccabi Health Services increased between 2011 and 2014. Although age-specific birth rates did not substantially change over this time period, the demographic change was accompanied by an overall decrease in live births after IVF treatment. The relative contribution of changing population demographics vs. current age-related coverage policies to these trends is unclear. Additional research is needed to better understand the potential effect of changes in current policy on maternal, neonatal, and economic outcomes.

## Background

Economic incentives and disincentives clearly play a role in couples’ decision making about having children. In the United States, birth rates declined during the Great Recession, with declines greatest in those states most affected by job loss [1]. Studies from Israel suggest that policies and practices affecting the level of child support are strongly correlated with birth rates [2, 3]. Not surprisingly, couples are more likely to try to have children when there is greater certainty that the resources needed to raise those children will be available.

For couples who are unable to spontaneously conceive, there is an additional consideration—the resources needed to undergo diagnosis and treatment of infertility. Again, there is evidence that infertility services are more likely to be utilized when those resources are at least partly covered by private or government insurance. Utilization of assisted reproductive technology (ART) varies between states in the U.S., with utilization greater in states where there is a legal mandate for insurers to provide coverage [4–6]. Increasing access in this way may have some beneficial effects—if the costs of multiple cycles is lower, couples may be less inclined to opt for more aggressive per-cycle treatment (such as replacement of multiple embryos) [4–6], which, while increasing the per-cycle success rate, also increases the probability of multiple gestations, with accompanying increased risk for maternal complications, preterm birth, and short- and long-term morbidity in the offspring.

Although there are a number of causes of infertility, a substantial proportion of couples will have no identifiable underlying pathological diagnosis; for many of these couples, the most likely reason for a decline in fecundity is the age-related decline in ovarian function in women. “Older” women make up an increasing proportion of the infertility population in many centers [7]; success rates in the absence of the use of donor oocytes in this population are substantially lower than for younger women [8].

## Commentary on Kol et al. [9]

In a recent IJHPR article, Kol and colleagues report on trends in the utilization and outcomes of in vitro fertilization (IVF) from 2007–2014 within the Maccabi Healthcare Services, which cover approximately 25 % of the Israeli population. The key findings of their report are:

- The total number of treatments increased from 2007 to 2011, with a subsequent decrease afterwards; this was accompanied by a decrease in overall live birth.
- The age distribution of the population undergoing IVF treatment changed from 2011–2014, with the proportion of women 25–39 (those with the highest success rates) declining from 67 to 57.6 %.
- Age-specific success rates, defined as live births divided by number of IVF cycles, were fairly consistent from year-to-year (Table 2 in Kol et al.).
- Live birth rates per cycle and per patient declined from 2007 to 2014, in parallel with the change in the demographics of the population.
- There is substantial variability in live birth rates between centers, although data on patient demographics and clinical aspects of treatment are not presented.

These data suggest that the overall decline in live birth rates observed in the population covered by Maccabi Healthcare Services is largely attributable to an increase in the proportion of IVF patients where the female partner is 40 or older. The authors attribute this phenomenon to Ministry of Health policy allowing relatively unrestricted access to multiple IVF treatments for women under the age of 45, leading to exceptionally high rates of IVF on a population-basis, but overall success rates substantially lower than the rest of the developed world [9].

Although the authors’ speculation about the relationship between unrestricted access to IVF and the observed outcomes is certainly plausible, there are some other factors to consider, especially with respect to this particular data set.

Although Maccabi Healthcare Services represents 25 % of the female Israeli population in the reproductive age groups [9], it is not necessarily the case that the relevant characteristics of this population in terms of age, distribution of infertility diagnoses, income, or other factors contributing to utilization and outcomes of infertility services are representative of the entire Israeli population. At the very least, it would be helpful to know how the age distribution compares to that of the overall Israeli population.

Second, it would be very helpful to know the population-based rate of IVF utilization, rather than just the raw numbers of cycles. Other countries, including those where coverage is much more variable, are also observing increased utilization of infertility services by older women [7]; some of the increase in raw numbers of treatments in older women may simply be attributable to increased numbers of older women, along with cohort effects associated with delayed childbearing. The argument that unlimited access to IVF treatment for older women with poor prognosis is an inefficient use of resources is an important one to make, but it would be useful to understand the extent to which increased numbers of IVF cycles in older women are due to changes in the numerator of the age-specific rate (increasing numbers of procedures among women in the 40–44 year age group for a given number of women in that age group), or in the denominator (similar numbers of procedures in women 40–44 for a given number of women in that age group, but the total number of women in the age group has increased). If nothing else, better understanding of the relationship between demand for services and the underlying demographics of the patient population would be useful for making estimates of the impact of changes in policy on IVF-related outcomes.

Third, there is a growing consensus that the most appropriate outcome for assessing the effectiveness of infertility treatment is the cumulative live birth rate per couple over time [10], which may result in much different conclusions compared to per cycle success rates. The data presented in the paper by Kol and colleagues make it difficult to draw conclusions about this metric. Although it is possible to calculate live births per couple treated, the distribution of the number of treatments per couple is not presented. In addition, it would be very helpful for the purposes of policy analysis to know whether decreased success rates in older women were also accompanied by increases in complications of treatment, or in increased rates of maternal or neonatal complications of pregnancy. Women 40 and older who have undergone infertility treatment are at increased risk for a number of complications, even with singleton pregnancies, although use of donor oocytes seems to eliminate most of this risk [8]. While the economic argument against unlimited access to treatments with a low probability of success is compelling, it would be even stronger if there were evidence that unrestricted access was leading to increased harms to mothers and infants.

## Conclusions

During the past five years, the proportion of IVF treatments provided by the Maccabi Healthcare Services to women aged 40–44 has increased substantially, which has been accompanied by an overall decrease in success rates per treatment cycle. The extent to which current Israeli Ministry of Health policies regarding age restrictions on IVF treatment are responsible for this observation, as opposed to changes in the underlying patient demographics, is unclear. Potential changes in coverage policies should consider estimates of the size of the potential patient population, trends in the epidemiology of different causes of infertility, both benefits (cumulative live birth per couple) and harms (maternal and neonatal complications) of IVF treatment in women of different age groups, and the economic impact of these policies.

## Abbreviations

ART, assisted reproductive technology; IJHPR, Israel Journal of Health Policy Research; IVF, in vitro fertilization
